# Biomarkers for Sepsis: A Review with Special Attention to India

**DOI:** 10.1155/2014/264351

**Published:** 2014-03-19

**Authors:** George E. Nelson, Vidya Mave, Amita Gupta

**Affiliations:** ^1^Division of Infectious Diseases, The Johns Hopkins University School of Medicine, 1830 East Monument Street Suite 450B, Baltimore, MD 21287, USA; ^2^BJMC Clinical Trials Unit, BJ Medical College and Sassoon General Hospitals, Jai Prakash Narayan Road, Pune 411 001, India; ^3^Center for Clinical Global Health Education, Johns Hopkins University School of Medicine, Baltimore, MD 21287, USA

## Abstract

Sepsis is a serious infection and still a common cause of morbidity and mortality in resource-limited settings such as India. Even when microbiologic diagnostics are available, bacteremia is only identified in a proportion of patients who present with sepsis and bloodstream infections. Biomarkers have been used in a variety of disease processes and can help aid in diagnosing bacterial infections. There have been numerous biomarkers investigated to aid with diagnosis and prognostication in sepsis with the majority suffering from lack of sensitivity or specificity. Procalcitonin has been heralded as the biomarker that holds the most promise for bloodstream infections. Data are emerging in India, and in this review, we focus on the current data of biomarkers in sepsis with particular attention to how biomarkers could be used to augment diagnosis and treatment in India.

## 1. Introduction

Sepsis and its complications are a common cause of infectious disease illness and mortality worldwide [1] and are a significant contributor to child death in India [2, 3]. Consensus definitions of sepsis were first published in 1992 [4] and later updated [5]. Better understanding of the pathophysiology of sepsis, new diagnostics, and improved therapeutics were reviewed in the surviving sepsis campaign guidelines [6] and subsequently revised [7]. International guidelines were published, and these have been supported and published in Indian medical journals [8].

Sepsis is defined as systemic inflammatory response syndrome (SIRS) caused by infection [7, 9]. However, infections can be difficult to confirm. Fever, tachycardia, hypotension, and other vital sign abnormalities found in SIRS are not specific for infection and overlap with noninfectious etiologies presenting with systemic inflammation. There is no gold standard for diagnosing infection, and though blood cultures processed with standard microbiologic techniques are a frequent diagnostic step, their likelihood of returning with the pathogen of interest depends on a variety of factors, including prior antibiotic therapy [10, 11]. Delays in empiric treatment for sepsis and bacteremia increase mortality [12] as well as length of stay [13] and cost [14], making timely recognition of infection and initiation of appropriate therapy an important goal. Standard blood culture techniques require time with results typically not available for at least 24–48 hours, highlighting the need for rapid diagnosis and risk stratification where biomarkers could be of use.

There have been many attempts to augment clinical decision making with diagnostic tests to increase sensitivity and specificity when diagnosing and treating sepsis and bacteremia. Initial studies employed fever and leukocytosis to define sepsis [4], though these tests were nonspecific. Subsequent studies focused on erythrocyte sedimentation rate (ESR) and C-reactive protein (CRP) to help in the diagnostic algorithm but suffered from the same lack of specificity. As our knowledge of sepsis evolved, it became evident that not only direct pathogen effects but also an exuberant inflammatory host response was responsible for the deleterious clinical and laboratory abnormalities. Sepsis is a systemic inflammatory syndrome affecting all organ systems, and biomarkers have focused on a number of pathogen and host responses, including cytokines, cell markers, receptor biomarkers, coagulation, vascular endothelial damage, vasodilation, organ dysfunction, acute phase protein markers, and other systems. Sepsis provokes a systemic host response involving hundreds of mediators that could be potentially used as biomarkers for both diagnosis and prognosis [15]. A recent review detailed nearly 180 biomarkers that have been evaluated including IL-6, IL-8, lactate, soluble triggering receptor expressed on myeloid cells-1 (sTREM-1), and procalcitonin (PCT) [16]. PCT has been the most studied and felt to hold the most promise.

India, with population of 1.2 billion [17], has one of the highest infectious disease burdens in the world [18]. While systemic data on presentations of acute febrile illness are lacking, 12% of adults (range 1–51%) of those presenting with acute febrile illness will have bacteremia [19]. While sepsis is not interchangeable with bloodstream infections, the majority of research has been done on sepsis as a syndrome and will be evaluated in this review. Availability of diagnostic assays is variable in India [20], making diagnosis of these common infections even more difficult. There is great interest in developing decision tools that utilize biomarkers to help aid the rapid diagnosis of bacterial infections. Additionally, due to rising antibiotic resistance on the Indian subcontinent, biomarkers that help with antibiotic stewardship are equally needed. There have been numerous studies evaluating PCT in different clinical scenarios, including sepsis, though the majority of these studies have been in the United States and Europe; there is great opportunity for well-designed studies evaluating biomarkers for sepsis in India.

## 2. WBC, ESR, Lactate, and CRP

Initial consensus statements focusing on sepsis definitions employed vital sign abnormalities and leukocytosis [4], but it is well recognized that overlapping with noninfectious etiologies exist [21]. Other routinely obtained, widely available tests such as lactate, serum glucose, and platelet counts that exhibit abnormalities in sepsis are nonspecific [9]. While leukocyte count was employed in initial definitions of sepsis, both leukocyte count and reliance on immature forms have low predictive value [22–24]. Lactate has been incorporated into definitions for sepsis, and normalization of serum lactate levels has been used as part of goal-directed care [25]. There is a broad consensus that an association between elevated lactate concentrations and poorer outcome is seen; however, a recent review [26] that included 28 studies found no ability to recommend a threshold value because of the extensive overlap of levels among patients with different outcomes. There is also the belief that elevated lactate levels occur later in disease and are less helpful as biomarkers from a diagnostic perspective because other signs, symptoms, or data will be available by that time. Serum lactate concentration at time of admission has been recommended by the surviving sepsis campaign guidelines as a marker of hypoperfusion [6] and a trial looked at using serum lactate to monitor resuscitation efforts [27].

ESR has long been used as an adjunctive test for inflammation; however, its utility as a biomarker for sepsis is limited [28]. There have been many studies comparing ESR with CRP; a recent study looked at the clinical utility of each test and variations in results stratified by age and concluded that each test provided similar information but that the time lapse for escalation and resolution was faster for CRP [29]. In this review, we will focus on CRP as it relates to sepsis. CRP is an acute-phase reactant produced only by hepatocytes in response to inflammation or tissue injury. In healthy young adult volunteer blood donors, the median concentration of CRP is below 0.8 mg/L and can increase 1,000-fold in response to an acute-phase stimulus [30, 31]. CRP hepatic synthesis starts rapidly after a stimulus with rise noted by about 6 hours with peak around 48 hours and a plasma half-life of approximately 19 hours. The half-life is constant under all conditions, so hepatic synthesis determines the serum concentration [32]. IL-6, as well as other cytokines, has been found to stimulate CRP production [31]. ESR and CRP have been known for a long time to be elevated in inflammatory conditions, including infection, and were used widely as an adjunctive test in sepsis and have often been used as a comparator for newer biomarkers [33]. Later studies have questioned their utility due to lack of specificity [34–36]. Studies have found that CRP changes were not influenced by neutropenia in septic patients [37], but CRP was not a good predictor of infection in neutropenic patients [38]. Elevated CRP levels in sepsis have been correlated with increased risk of death and organ failure [39], but in part due to the persistence of elevated levels, were unable to predict survival when evaluating CRP trends [40, 41]. CRP has been used successfully during initial sepsis diagnosis, but its specificity is further reduced later in the course due to persistently elevated levels [42]. CRP has been found to be significantly elevated in sepsis due to gram negative infections compared with gram positive infections suggesting a different immunomodulatory response [43].

A year-long study evaluating 57 episodes of febrile neutropenia among 26 young adults found that a rise in CRP on day 3 showed a significant difference between those with microbiologically defined infection when compared with fever without microbiologic diagnosis and was able to differentiate those that responded to addition of antifungal therapy and those that responded to second line antimicrobial therapy [44].

There have been several investigations of CRP in sepsis in India. Sugitharini et al. found that CRP levels were significantly elevated in neonates with sepsis compared with those without [45]. While Sugitharini's study did not report sensitivity of CRP in detecting sepsis, a study comparing CRP levels in 80 septic pediatric patients in India with 30 healthy pediatric controls found that CRP had a sensitivity of 67%, specificity 97%, PPV 98%, and NPV 53%; this study found a higher sensitivity and specificity for TNF-alpha levels (sensitivity 85%, specificity 100%, PPV 100%, and NPV 71%) [46]. While not specific to bacteremia, malaria is a common complicating factor in patients who appear septic in India which is not seen in locations that the majority of biomarker research has been conducted; researchers have found that CRP is elevated in cases of acute malaria [47] and degree of CRP elevated was correlated with death and length of hospitalization. CRP levels are known to be influenced by genetic variants in Europeans, and one study evaluated genetic variants in Indian patients [48]. A study looking at nondiabetic Asian Indians living in the United States found significant elevation of plasma CRP levels [49]. More research will need to be done to see how this affects interpretation of CRP in Indian patients, though CRP will likely not be found to have the necessary discriminatory power for diagnosis and treatment of sepsis.

## 3. Procalcitonin

Procalcitonin is a 116 amino acid polypeptide precursor for the hormone calcitonin. It was first identified in 1975 and first linked to infectious disease in 1983 when increased serum levels of immunoreactive calcitonin were described in patients with staphylococcal toxic shock syndrome [50]. It was not until Assicot et al. reported high serum PCT levels in sepsis that the current research on PCT in bacterial disease accelerated [51]. Procalcitonin offers favorable kinetics for a biomarker: rising prior to two hours [52], reliably detectable between 2 and 4 hours, peaking at 6 hours, and maintaining a plateau through 8 and 24 hours [53]. At physiologic homeostasis, PCT is detectable in very low levels in the serum in healthy individuals [54] and can increase 1000-fold during active infection. During infection in an animal model, PCT is released from many cell types distributed throughout the body [55] and is induced by interleukin-1*β*, tissue necrosis factor (TNF)-*α*, IL-6, and lipopolysaccharides and can be attenuated by interferon-*γ* that is elevated during viral infections [56]. These and other observations have led to the extensive evaluation of PCT as a marker of sepsis and bloodstream infection.

While interpretations of many biomarkers suffer from elevations in conditions other than bacterial infection, PCT has shown promise in improved specificity in bacterial infections. Early studies showed that PCT showed differences in infectious versus noninfectious, inflammatory conditions [57–59]. It has been shown to be able to differentiate between patients with confirmed bacterial* versus* viral infections with high sensitivity (95%) [60]. It has also been used to evaluate secondary bacterial superinfection in patients admitted with influenza [61, 62]. PCT had high sensitivity to exclude bacteremia in urosepsis [63] and community acquired pneumonia [64]. Additionally, there have been studies showing that bacteremia is highly unlikely when PCT levels are below the threshold 0.1 ng/mL [65].

There have been several meta-analyses evaluating PCT as a diagnostic marker in sepsis [33, 66–68]. While the earlier meta-analyses had conflicting results and were limited by populations studied and sepsis definitions, the most recent meta-analysis [68] evaluating 30 studies with 3244 patients yielded a sensitivity of 77% (95% confidence interval (CI): 72–81%) and specificity of 79% (CI: 74–84%) indicating that it was a useful biomarker for diagnosis of early sepsis, but could not be used in isolation and must be interpreted in context of patient presentation.

Several studies have evaluated PCT for diagnosis of sepsis in an emergency department (ED) setting. A meta-analysis published in 2007 including 17 studies found a sensitivity of 76% and specificity of 70% for the detection of sepsis; however, these studies were heterogeneous in the prevalence of sepsis and PCT cutoffs used for diagnosis [69]. A more recent study evaluated 336 adult emergency room patients of which 60% had definite infection; PCT levels were higher in septicemia (median PCT 2.3 versus 0.2 ng/mL) and concentrations increased with likelihood of infection and sepsis severity [70]. PCT best predicted septicemia when compared with IL-6 and CRP with 73% sensitivity and 70% specificity for bacteremia with a cutoff of 0.5 ng/mL.

PCT levels have been found to differ between medical and surgical patients with septic shock with higher baseline levels in surgical patients proposed to be due to transient bacteremia, endotoxin release, or ischemia, and a higher threshold value (9.7 ng/mL) had higher sensitivity (92%) for surgical patients [71]. PCT levels have also found to differ for neonatal patients with sepsis with a meta-analysis showing that neonates with sepsis and meningitis sensitivity ranged from 81 to 100% [72].

PCT does not appear to be affected by neutrophil count and has been evaluated in patients presenting with neutropenic fever. A recent meta-analysis of 30 studies evaluating PCT in neutropenic patients found PCT to be helpful, but not diagnostic for bacteremia due to lack of standard definitions, heterogeneity of study populations, and small number of patients included in some of the studies [73]. Knowing that PCT levels increase with severity of infection and over time, serial levels may be indicated in this population. It should be noted that PCT remains unaffected by corticosteroids when compared with CRP [74].

Data are not sufficient to make determinations of PCT use in fungemia. PCT levels in candidemia do not appear to show the same level of elevation as in bacteremia; one retrospective analysis of bacteremia and candidemia in nonneutropenic patients showed a significantly lower PCT level in candidemic patients [75]. However, most of the studies evaluating PCT in invasive fungal infections are limited by small case counts; a recent meta-analysis including 8 studies with 474 episodes of suspected infection (155 confirmed or probable invasive fungal infections) showed a pooled sensitivity of 0.82 (95% CI, 0.48–0.95) and specificity of 0.80 (95% CI, 0.60–0.91) [76]. They noted the negative likelihood ratio could not be used to safely exclude systemic fungal infection. It should be noted that the studies included had a wide range of PCT cutoffs (0.3–5.5 ng/mL).

PCT has been investigated in several studies in India, though most have focused on case reports or series looking at specific diagnoses such as scrub typhus [77], septic arthritis and osteomyelitis [78], H1N1 [79], pancreatitis [80, 81], pyelonephritis [82], and meningitis [83].

One Indian study looked at PCT with a semiquantitative PCT test as well as eubacterial PCR in comparison with blood cultures [84]. Ninety patients (60 septic patients compared with 30 nonseptic patients) were evaluated; compared with blood cultures, the sensitivity, specificity, and positive and negative predictive values for PCT were 100%, 62%, 57%, and 100%, respectively. The authors concluded that PCT may be useful as a rapid test for detecting septicemia but compared with blood cultures lacked specificity which may be in part to the high cutoff value of 2 ng/mL that was used in this study. A more recent prospective study in India conducted from 2006 to 2008 evaluated 100 patients and found a sensitivity of 94% with a significant association with Sequential Organ Failure Assessment scores, but no significance for severity of sepsis or mortality [85]. Another study conducted in an Indian ICU setting evaluated 40 patients found that patients with PCT ≥ 2 ng/mL had statistically significant correlation with the presence of sepsis (*P* < 0.0001) with a moderate sensitivity (86%) and high specificity (95%) [86]. It should be noted that a 2 ng/mL threshold is higher than many studies (0.1–2 ng/mL). PCT was evaluated as a biomarker in neonatal sepsis in 118 neonates with early onset sepsis compared with 61 normal samples [45]. There were significantly higher levels of PCT (1.500 ± 0.2400 *μ*g/L)  in  neonates with sepsis. Obviously, there is great opportunity to study PCT in India.

## 4. PCT for Antimicrobial Stewardship

Due to its ability to help differentiate between viral and bacterial infections, PCT has been evaluated for its ability to guide decisions for appropriate antibiotic therapy. India has one of the highest rates of infectious diseases and has alarmingly high rates of resistant bacteria, making utilization of diagnostics that help indicate when unnecessary antibiotics can be avoided a prime goal [87, 88]. Initially there were two small, single center studies investigating the use of PCT in an antibiotic management algorithm for septic patients. The first evaluated serial PCT measurements in 39 patients compared with 40 controls in an ICU setting and found a 4-day reduction in the duration of antibiotic therapy (*P* = 0.003) and a smaller overall antibiotic exposure (*P* = 0.0002), 2-day shorter ICU stay (*P* = 0.03) without a difference in 28-day mortality, clinical cure, or relapse [89]. The second evaluated surgical ICU patients and found a decrease in antibiotic exposure days (5.9 ± 1.7 versus 7.9 ± 0.5 days, *P* < 0.001) and decrease in length of stay without negative effects on outcomes [90]. The PRORATA trial, a multicenter, prospective, open-label, and randomized control trial including 621 patients in 8 ICUs in 6 hospitals followed these smaller studies and found a 23% reduction in antibiotic usage at day 28 [91]. Importantly, PCT guided deescalation in antibiotics was noninferior to standard of care with a 10% noninferior mortality difference assumed. They cautioned not to extrapolate results to surgical ICU patients as they comprised only 10% of the population and concluded that PCT-based algorithms are likely to have the greatest benefit at aiding discontinuation of antibiotics rather than withholding them from critically ill patients especially given that no ideal threshold for starting or withholding antibiotics in critically ill patients has been established. PCT measurements have also been found to be statistically significantly higher in patients with true bacteremia when compared to patients deemed to have contaminants with coagulase negative staphylococci [92] which certainly have implications for decreasing inappropriate antibiotic use. There have been numerous other studies evaluating deescalation of antibiotics in a variety of clinical syndromes, including respiratory disease [93].

There have been several meta-analyses of PCT algorithms that evaluated antibiotic use. Three evaluated a variety of infections [94–96]. The first included 14 randomized controlled trials (RCTs) (*N* = 4467 patients) that investigated PCT algorithms for antibiotic treatment decisions in adult patients with respiratory tract infections and sepsis from primary care, ED, and ICU settings. There was no difference in mortality in any setting. In the primary care setting, 2 studies found 74–42% reduction in antibiotic prescription and a 13–0% [97, 98]; in the ED 6 trials found reduction in antibiotic prescription from 47 to 15% and duration from +8% to −55% [99–101]; in the ICU setting 6 trials showed reduction in duration from 37 to 20% [89, 102]. The second review [95] evaluated 6 published RCTs comparing PCT-guided antimicrobial therapy to usual care in ICU patients; PCT guidance was associated with significantly reduced antimicrobial exposure (effect sizes, 20%–38%). The third [96] included 7 studies with 1458 patients (4 with respiratory infections, 2 with septic patients, and 1 with surgical ICU patients) and found that PCT-guided therapy was associated with reduction in antibiotic use at inclusion (4 studies, pooled OR 0.506, 95% CI 0.290–0.882, *P* = 0.016), duration of antibiotic therapy (6 studies, weighted mean difference (WMD) 2.785, 95% CI 1.225–4.345, *P* = 0.000), and total antibiotic exposure days/1,000 days (4 studies, pooled RR 1.664, 95% CI 1.155–2.172, *P* = 0.000) without differences in length of total hospital stay or mortality.

Four meta-analyses [103–106] evaluated PCT-guided treatment on antibiotic usage in ICU patients. Heyland et al. found that PCT-guided treatment was associated with a significant reduction in antibiotic use (WMD −2.14 days, 95% CI: −2.51 to −1.78, *P* < 0.00001) [103]. Another meta-analysis [104] included 7 RCTs with 1131 ICU patients and found that use of PCT-guided strategies decreased the duration of antibiotic therapy for the first episode of infection (WMD −2.36 days, 95% CI: −3.11 to −1.61) and the total duration of antibiotic treatment by 4 days (WMD −4.19 days, 95% CI: −4.98 to −3.39) without difference in 28-day mortality or relapse infection rate. Another analysis [105] incorporated 7 studies, 6 of which were included in the previous meta-analysis [104] and found similar decreases for antibiotic use for first infection episode (pooled WMD = −3.15 days, 95% CI: −4.36 to −1.95, *P* < 0.001) and no difference in mortality. The most recent meta-analysis by Prkno et al. [106] was the first to analyze PCT-guided treatment effects on antibiotic use and clinical outcomes in ICU patients with severe sepsis and septic shock. In that analysis 7 studies consisting of 1075 patients observed that while mortality and length of stay were no different between PCT-guided treatment and standard of care, there was a statistically significant decrease in duration of antibiotic use in the PCT-guided approach (hazard ratio: 1.27, 95% CI: 1.01; 1.53). They comment that using a PCT-guided approach for treatment of severe sepsis reduces antibiotic exposure without an obvious difference in mortality, though more research is needed to further define the PCT algorithms used in different patient populations as different cutoff values were used for different patient populations with differences noted between medical and surgical patients with severe sepsis.

Kaur et al. published a review in 2013 evaluating PCT in an effort to reduce inappropriate antibiotics in an Indian emergency setting [107], but no studies evaluating PCT for antibiotic stewardship in India have been published to date.

## 5. PCT Assays, Cost, and Implementation in India

Currently there are several PCT assays that have been developed and compared in the literature. The main manufacturer of PCT assays is BRAHMS and include the Kryptor, VIDAS, PCT-Q, and PCT LIA assays [108].

PCT sensitive Kryptor assay provides a sensitive, functional assay sensitivity of 0.06 ng/mL with results available in 19 minutes using 20–50 mL of plasma or serum [109]. The BRAHMS PCT LIA is a manual PCT assay that can test plasma or serum using a luminometer and has a detection limit of ~0.3 to 0.5 ng/mL with results available after 1 hour incubation time [110]. The LIASON BRAHMS PCT is a fully automated random access analyser that uses two-site immunoluminometric assay with functional sensitivity of 0.3 ng/mL and can have results within 30 minutes [108]. VIDAS BRAHMS PCT is an enzyme-linked fluorescent immunoassay providing quantitative PCT measurements with a functional detection limit of 0.09 ng/mL [108]. The BRAHMS PCT-Q is a manual immunochromatographic test for the semiquantitative detection of PCT after incubation of 30 minutes and can distinguish ranges above 0.5 ng/mL [108]. The PCT-Q is marketed as a point-of-care testing kit. Results are indicated by four different shades of red, corresponding to different PCT ranges, indicating the possibility and severity of sepsis [111]. Although the kit is designed to require no specialized training, the semiquantitative nature requires interpretation by the operator, and user difficulties in interpreting results have been reported, and its results only showed moderate agreement compared with the Kryptor assay when used in the clinical setting [111]. There are several other PCT platforms available from BRAHMS.

Schuetz et al. reported that the Kryptor and VIDAS systems could be used interchangeably [112], and Steinbach et al. found agreement between the Kryptor and PCT-Q systems for ranges of PCT that were common to both systems [113]; PCT-Q assay has the disadvantage of being able to discriminate values <0.3 ng/mL. The fully automated LIASON system has been found to have good correlation with the previous PCT-Q assay as well [114].

While purchasing a PCT platform can be expensive, there have been several analyses to indicate that PCT-based algorithms might be cost-effective in different patient populations and illnesses [103]. Heyland et al. performed an economic analysis of PCT-based algorithms compared with standard of care in a meta-analysis [103] including five studies, four of which have been referenced earlier in this paper [89–91, 102]. As the results of the analysis demonstrated no difference in mortality, length of stay, or recurrent infection, cost analysis in Canadian dollars evaluated acquisition costs of antibiotics, administration costs of intravenous antibiotics, and PCT testing costs, including assay material, reagents, technician time, purchase, maintenance of a bench top analyzer, and overhead. They utilized three cost scenarios and found an average cost savings per treatment episode of Can$470.62 in 2011. This number increased to over Can$1100 cost saving per episode using more expensive antibiotics but showed an increase in cost over standard therapy by Can$193.64 per patient using the least expensive antibiotic scenario and most frequent PCT testing algorithm. Total cost savings depend on a variety of factors including local costs of the PCT assay, the frequency of PCT measurement, and the cost and duration of the antibiotics used.

It is difficult to translate the above data to India where antibiotic use, availability, and ease of implementing a test are drastically different. As cost changes over time, it is always difficult to use historical studies such as Heyland's which was published in 2011. But as PCT testing becomes more mainstream, testing cost will decrease while still providing opportunity to decrease antibiotic use. As semiquantitative testing, such as the PCT-Q test, is the least expensive and the easiest to implement and provides point-of-care results, this may be the platform of interest until other options are available. The area of cost-effectiveness of PCT testing in sepsis in India is an opportunity for further research.

## 6. sTREM-1, IL-6, IL-8, IL-27

While PCT has shown the most promise and has been the most studied of biomarkers for sepsis and bloodstream infections to date, there are hosts of other biomarkers that have been evaluated with a recent review indicating at least 180 that have been researched [16]. Triggering receptor expressed on myeloid cells-1 (TREM-1) was reported to be upregulated in various inflammatory diseases as well as in sepsis; TREM-1 expression is associated with elevations in soluble TREM-1 (sTREM-1). Studies have shown that the expression of TREM-1 is elevated* in vitro* in the presence of bacteria or fungi as well as peritoneal fluid and tissue from infected patients [115, 116] but remains at normal levels in noninfectious inflammatory conditions and may be a therapeutic target for sepsis [117].

Some studies have shown sTREM-1 to be superior to CRP and PCT [118] but other studies have shown that sTREM-1 has poor discriminatory power compared with routinely available parameters [119]. A recent meta-analysis found that the sensitivity of sTREM-1 for the diagnosis of bacterial infection was 82% and that the specificity was 86% [120].

While IL-6 and IL-8 levels have been shown to be elevated in sepsis and associated with severity and outcome [121], they have not been found to be superior to PCT as biomarkers [58, 70]. These cytokines have been found to be elevated in neutropenic fever [38] and neonatal sepsis [122] but have been less useful in the adult population [123].

Wong and colleagues looked at genome-wide transcriptional profile differences in leukocytes between infected and noninfected pediatric ICU patients and found 221 differentially expressed probes [124]. Individual patient mosaics were assigned to either noninfectious illness or sepsis classes thereby achieving 90% specificity and 94% PPV. Interleukin-27 (IL-27) presented the highest predictive power. The same group subsequently validated their findings by measuring serum levels of IL-27 in a separate study and found serum IL-27 concentrations were significantly higher in patients with sepsis in comparison with noninfected patients yielding 92% specificity and 91% PPV for bacterial infection in critically ill children. There are many more candidate biomarkers that have been developed that require more investigation prior to use, including markers of endothelial cell activation [125].

Data in the Indian population on these biomarkers are lacking as a whole. Sugitharini also looked at a variety of mediators in neonatal sepsis [45] including granule-associated mediators (neutrophils elastase (NE), myeloperoxidase (MPO), and nitric oxide (NO)), proinflammatory cytokines (TNF*α*, IL-1*β*, and IL-6), anti-inflammatory cytokines (IL-10 and IL-13), chemokines (IL-8 and monocyte chemotactic protein (MCP-1) and novel cytokines). They found significantly higher levels of NE, NO, TNF*α*, IL-1*β*, IL-6, and IL-8 in neonates with early onset sepsis compared with controls. The levels of MPO were downregulated, and there was no change in IL-13. The presence of 17 inflammatory proteins including IL-16, TNF*α*, TNF*β*, and MCP-1 were upregulated in neonatal sepsis. This study evaluated a number of biomarkers that are dysregulated during sepsis in a neonatal population suggesting that many of the ideas about biomarkers in studies in Europe and in adult patients may be similar after more study is done.

## 7. Future Directions

The many biomarkers that are under investigation for sepsis diagnosis and prognosis have been well documented, but no one test is sufficient to exclude bloodstream infection. There has been hope that a combination of biomarkers could create a useful algorithm with adequate sensitivity and specificity to aid in diagnosis.

Initial studies attempted to incorporate PCT into decision models and found that CRP improved model fit and created a resulting score that was more accurate than physician judgment of SIRS alone [126]. Utilizing data from the expanding research on biomarkers, an observational study evaluated 17 immune mediators and employed a combined cytokine score with IL-6, IL-8, and IL-10 and showed it was useful predictor of outcome [127]. A study of 151 patients (96 with bacterial infections) were evaluated with 6 biomarkers including sTREM-1, CRP, and PCT and found that a combination of the markers showed improved diagnostic ability compared with any single maker [128]. Another approach that created a bioscore using PCT, sTREM-1, and CD64 index in 300 consecutive patients and subsequently externally validated the score in an independent prospective cohort of 79 patients found each biomarker to be independent predictors of infection but the performance of the bioscore to be superior to each individual biomarker and significantly elevated (*P* < 0.001) in patients with sepsis compared to noninfected patients [129].

Combination of biomarkers has also been evaluated in a pediatric sepsis model [130]. After a genome-wide expression profiling, a risk stratification tool investigated 12 markers and employed 5 biomarkers in the analysis. The PERSEVERE model was found to have sensitivity for mortality of 93% (79–98), specificity 74% (69–79), PPV 32% (24–41), and NPV 99% (96–100).

Combination models have also been evaluated in neonatal sepsis evaluating four tests (microerythocyte sedimentation rate, immature to total neutrophil count, morphological changes in neutrophils, and CRP) and found the role of these tests in early diagnosis of neonatal sepsis were statistically significant (*P* < 0.05) with a combination of three or all of these four tests was highly specific (95–100%) [131].

In order to advance the field of biomarker research in India, well-designed studies are necessary to evaluate threshold values for the diagnosis and deescalation of antibiotics in Indian patients with sepsis. Additionally, more study of specific subgroups, including pediatric versus adult patients, varying severity of sepsis, medical versus surgical patients, and other populations with specific syndromes is needed in general, and in India, in specific. Further study of investigational biomarkers that may hold promise for evaluating sepsis either as an individual test or in conjunction with other tests to improve sensitivity and specificity needs to be investigated for their potential use in India.

## 8. Summary

Sepsis continues to be a significant cause of morbidity and mortality despite advances in therapeutics and diagnostics. Biomarkers for sepsis, and by extension bloodstream infections, hold much promise for increasing the rapidity with which sepsis is diagnosed and for risk stratification for prognostication. Despite extensive research, no single biomarker can yet serve as the lone diagnostic parameter. PCT remains the most researched and utilized biomarker for sepsis. While cost effectiveness analyses have been done on PCT in acute respiratory infections, there is still a need for robust cost effective analyses in sepsis [132] which will be of keen interest in India to determine potential rational implementation strategies. While there are publications that come out of India evaluating PCT and other biomarkers in sepsis, the level of evidence is still not such to make definitive recommendations for use. PCT may be an effective tool for utilization in an algorithm for diagnosing sepsis and lessen dependence on microbiology resources that can vary in India. Data about biomarkers and PCT in sepsis are gradually increasing and will help provide informed next steps for research in India.
